# Disturbance of Gut Bacteria and Metabolites Are Associated with Disease Severity and Predict Outcome of NMDAR Encephalitis: A Prospective Case–Control Study

**DOI:** 10.3389/fimmu.2021.791780

**Published:** 2022-01-03

**Authors:** Xue Gong, Yue Liu, Xu Liu, Aiqing Li, Kundian Guo, Dong Zhou, Zhen Hong

**Affiliations:** ^1^ Department of Neurology, West China Hospital, Sichuan University, Chengdu, China; ^2^ Institute of Brain Science and Brain-Inspired Technology of West China Hospital, Sichuan University, Chengdu, China; ^3^ Department of Neurology, Chengdu Shangjin Nanfu Hospital, Chengdu, China

**Keywords:** NMDAR encephalitis, microbiome, metabolomics, prognosis marker, multi-omic analysis

## Abstract

**Objective:**

We aimed to investigate the associations between the intestinal microbiota, metabolites, cytokines, and clinical severity in anti-*N*-methyl-d-aspartate receptor (NMDAR) encephalitis and to further determine the predictive value of the intestinal microbiota or metabolites in clinical prognosis.

**Methods:**

In this prospective observational cohort study of 58 NMDAR encephalitis patients and 49 healthy controls, fecal microbiota, metabolites, and cytokines were quantified and characterized by16S rRNA gene sequencing, liquid chromatography–mass spectrometry, and the Luminex assay, respectively.

**Results:**

There were marked variations in the gut microbiota composition and metabolites in critically ill patients. We identified 8 metabolite modules (mainly characterized by fatty acid, glycerophosphoethanolamines, and glycerophosphocholines) that were distinctly classified as negatively or positively associated with bacterial co-abundance groups (CAGs). These CAGs were mainly composed of *Bacteroides*, *Eubacterium_hallii_group*, *Anaerostipes*, *Ruminococcus*, *Butyricicoccus*, and *Faecalibacterium*, which were substantially altered in patients. In addition, these fecal and serum metabolic modules were further correlated with the serum cytokines. Additionally, the combination of clinical features, microbial marker (*Granulicatella*), and a panel of metabolic markers could further enhance the performance of prognosis discrimination significantly, which yielded an area under the receiver operating characteristic curve of (AUC) of 0.94 (95%CI = 0.7–0.9). Patients with low bacterial diversity are more likely to develop relapse than those with higher bacterial diversity (log-rank *p* = 0.04, HR = 2.7, 95%CI = 1.0–7.0).

**Interpretation:**

The associations between the multi-omics data suggested that certain bacteria might affect the pathogenesis of NMDAR encephalitis by modulating the metabolic pathways of the host and affecting the production of pro-inflammatory cytokines. Furthermore, the disturbance of fecal bacteria may predict the long-term outcome and relapse in NMDAR encephalitis.

## 1 Introduction

Anti-*N*-methyl-d-aspartate receptor (NMDAR) encephalitis is the most prevalent and severe autoimmune encephalitis type, with significant economic burden (1, 2). Nearly 75% of patients may require care in the intensive care unit, and 20%–25% of cases may have poor prognosis or further relapse (3, 4). However, there are no biomarkers to inform therapy or predict outcomes. Despite some clinical signs (including orofacial dyskinesia and central hypoventilation) (3), higher antibody titers in the cerebrospinal fluid (5), increased cytokines (CXCL13, TNF-α, IL-6, or IL-10) (6, 7), or abnormal MRI (8, 9) suggesting potential associations with poor clinical outcomes, these associations were weak and need longitudinal assessments. Thus, biomarkers are still urgently needed for the prediction of disease prognosis and evaluation of disease severity in NMDAR encephalitis.

The microbiota could modulate the host immune response and affect the secretion of cytokines and antibodies (10–13). It has been reported that dysbiotic microbiota is involved in the pathogenesis of various antibody-mediated diseases such as multiple sclerosis, neuromyelitis optica, Guillain–Barré syndrome, and systemic lupus erythematosus (14–17). One leading hypothesis is that molecular mimicry leads to cross-reactivity (18, 19) and, in turn, to autoimmune attack. In our prior study, an increase of pro-inflammatory fecal bacteria was also observed in NMDAR encephalitis patients with several relapses (20). In addition, *via* fecal transplantation, one study reported that transplantation of the “NMDAR encephalitis microbiota” into specific pathogen-free (SPF) mice can induce abnormal behaviors and T helper 17 (Th17) response (21). Taken together, these studies have provided initial proof that microbial dysbiosis or imbalance may potentially contribute to the onset of this disorder.

Metabolomics is a new method for elucidating the pathomechanisms and biomarkers and for assessing the environmental impact of a disease. Metabolome (serum and fecal) also represent end products from microbial metabolism, and they are functionally more important when compared to specific bacterial species (22, 23). Therefore, exploring links between the microbiota and metabolic and immune function may reveal new insights into disease etiology and pathophysiology. However, as far as we know, no data are currently available for investigating the molecular mediators of the effects of the microbiota on NMDAR encephalitis. Furthermore, research on whether altered microbiota and metabolites are associated with or could predict the risk of susceptibility to severe status and worse outcomes in NMDAR encephalitis has not been determined yet.

Herein, for the first time, we present a comprehensive comparison of the intestinal microbiota and systemic metabolomes from a large and prospective cohort of 107 individuals. We first established the features of the gut microbiota and the host metabolite profiles that are correlated with disease severity. We then integrated extensive multilevel omics findings to elucidate the gut microbial ecosystem, and its significance, in NMDAR encephalitis. In addition, the predictive value of the intestinal microbiota or metabolites in the clinical prognosis of NMDAR encephalitis was revealed.

## 2 Methods

### 2.1 Study Design and Human Participants

**Figure 1:**
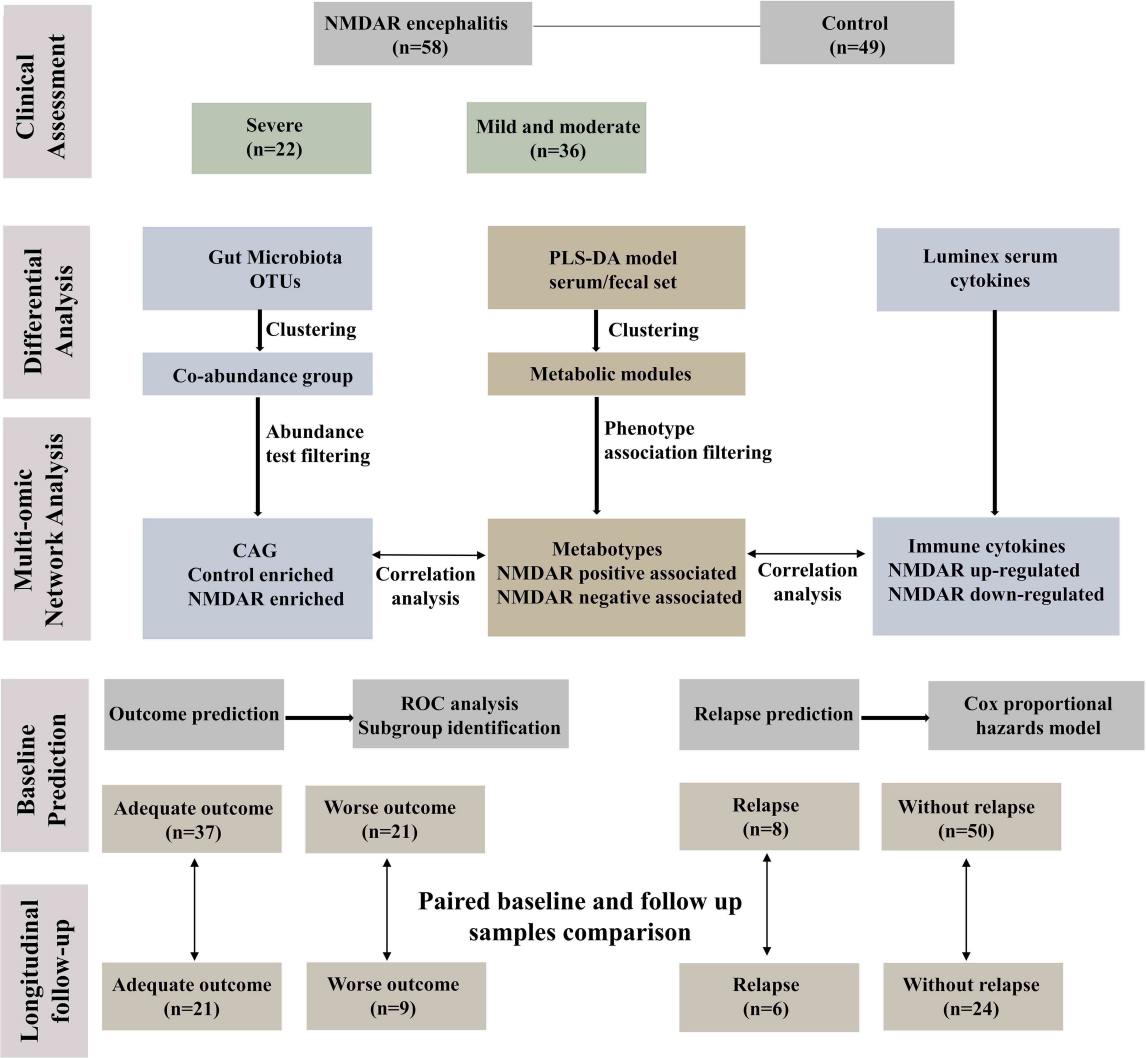
Workflow integrating anti-*N*-methyl-d-aspartate receptor (NMDAR) encephalitis gut microbiome, fecal and serum metabolomes, and cytokines. Fifty-eight patients with a definite diagnosis of NMDAR encephalitis were divided into different severity groups: severe (*n* = 22) and moderate (*n* = 36); there were 49 healthy controls. We next constructed a co-abundance network with 294 operational taxonomic units (OTUs) and clustered them into 19 co-abundance groups (CAGs). Subsequently, we identified the important CAGs that were strikingly prevalent in patients with more severe status. We then identified the serum and fecal metabolome and cytokine features between NMDAR encephalitis patients and healthy controls. Serum and fecal metabolites were summarized as co-abundance metabolic modules. Next, we identified the relationships between the markedly altered gut microbiome composition, host metabolic profiles, and the major dysregulated cytokines. Finally, fecal and serum samples from 30 patients after 6 months of follow-up were collected to investigate longitudinal deviations in dysbiosis and establish associations between disease outcome markers and dysbiosis.

**Figure 1** depicts the overall design of the study. Briefly, fecal specimens from a prospective cohort of 58 treatment-naive NMDAR encephalitis patients and 49 household healthy controls (HCs) were evaluated using high-throughput 16S ribosomal DNA (rDNA) gene sequencing and untargeted metabolomics. Serum samples were collected from a subset of these same NMDAR encephalitis patients (*n* = 39) and HC individuals (*n* = 31) for untargeted metabolic and cytokine study. All patients were followed for at least 6 months, after which the fecal and serum samples of 30 patients were collected at the remission phase.

All participants in this study were enrolled between December 2019 and March 2021 from West China Hospital of Sichuan University, in which treatment-naive patients with unknown triggers at initial onset (*n* = 58) had been diagnosed based on the definitions of NMDAR encephalitis from a recent consensus statement (1). The detailed diagnostic criteria of NMDAR encephalitis patients are summarized in *eMethods* in the *Electronic Supplementary Material* (ESM). We included spouse (*n* = 30), sibling (*n* = 14), or parents (*n* = 5) as controls free from neurological disorders living within the same community and sharing a similar diet for at least 5 years (to minimize the potential effects of confounding by factors, including diet, housing conditions, and lifestyle) (24, 25).

The exclusion criteria for HCs and patients were: 1) patients with identified trigger for disease (tumor or herpes simplex virus encephalitis); 2) participants who received treatment with any immunotherapies (steroids, immunoglobulin, cyclophosphamide, rituximab, or plasma exchange); 3) participants who received antibiotics or probiotics, or prebiotics within 1 month before sampling; 4) participants who experienced marked bloating, abdominal pain, diarrhea, or infections of the respiratory tract in the previous month; 5) alcohol or any other substance (apart from tobacco) dependence or abuse within 3 months before enrollment; 6) participants with chronic diseases such as hypertension and diabetes; 7) history of other autoimmune diseases (systemic lupus erythematosus, rheumatoid arthritis, type 1 diabetes, etc.); 8) history of bowel surgery; 9) lactation or pregnancy; and 10) any history of neuropsychiatric disorders, such as depression, schizophrenia, anxiety, or multiple sclerosis.

This study was conducted in accordance with the Declaration of Helsinki and was granted permission by the Institutional Review Boards of the Ethics Committee of West China Hospital of Sichuan University. Prior to their enrolment, all participants provided informed consent (registration no. ChiCTR2100042215; Chinese Clinical Trial Registry, http://www.chictr.org). The patient cohort included in this study was independent from our previous cohorts (20).

### 2.2 Clinical Evaluations and Follow-Up Assessment

Participants were subjected to various clinical evaluations, such as medical history, physical and neurological examinations, laboratory assessments, and neuropsychological tests. All clinical information [demographic, medical, follow-up, and body mass index (BMI) data] was collected according to standard procedures (see details in *eMethods* in ESM). Assessment of dietary intake was done using a locally validated Food Frequency Questionnaire (FFQ) including 136 food items and analyzed by a competent nutritionist, as previously reported (20, 26).

The modified Rankin scale (mRS) score was employed to assess disease severity and outcome grading of NMDAR encephalitis. The initial severity status was classified according to mRS scores of moderate (0–3) and severe (4–5). The respective definitions of long-term favorable or poor functional outcomes were as follows: mRS score of ≤2 and mRS score of >2 at 6 months after admission (27–29). Patients were routinely followed up every 2 months for up to at least 6 months. The primary outcome was the long-term outcome. The secondary end point was clinical relapse, which was defined as a new symptomatic onset or worsening after an initial stabilization or improvement for ≥2 months (1).

### 2.3 Specimen Collection and Quality Control

Determination of the sample sizes was based on previous studies (20). The participants were given a written protocol for stool collection (to avoid contamination of the sample) and transportation. Briefly, disposable sterile potty and tubes were distributed to participants in advance. Firstly, participants discharged their feces into the sterile potty, washed their hands, wore disposable gloves, took the middle part of the feces, instantly put them on ice, and transported the sample to the laboratory, where it was stored it at −80°C within 1–2 h after sample collection.

Serum samples were collected from patients who consented to participate in the study. For each participant, one 6-ml overnight fasting peripheral blood sample was obtained in the morning of the day after admission. Blood was centrifuged at room temperature for 10 min at 3,000 × *g*, then the serum was collected, and 1–2 ml aliquots were transferred into pre-labeled cryovials. Within 1 h of collection, the samples were kept at −20°C for 24 h for freezing and then stored at −80°C for further analyses.

### 2.4 DNA Extraction, Amplification, and Sequencing

Fecal samples stored at −80°C were used in this assay, which was performed at Majorbio BioPharm Technology Co., Ltd. (Shanghai, China). Extraction of microbial DNA from fecal samples was conducted using the E.Z.N.A.^®^ Soil DNA Kit (Omega Bio-Tek, Norcross, GA, USA) as described by the manufacturer. The final DNA concentrations were determined with a NanoDrop 2000 UV–vis spectrophotometer (Thermo Scientific, Wilmington, DE, USA), while DNA quality was evaluated with 1% agarose gel electrophoresis. Specific primers (806 R: 5′-GGACTACHVGGGTWTCTAAT-3′; 338 F: 5′-ACTCCTACGGGAGGCAGCAG-3′) were used to amplify the V3–V4 hypervariable regions of the bacterial 16S rRNA gene *via* a thermocycler PCR system (GeneAmp 9700; ABI, Foster City, CA, USA). The PCR conditions were as follows: 3 min of denaturation at 95°C, 27 cycles at 95°C for 30 s, annealing at 55°C for 30 s, elongation for 45 s at 72°C, and a final extension for 10 min at 72°C. The PCR assays were conducted in triplicate in a 20-μl mixture of 4 μl 5× FastPfu Buffer, 2.5 mmol/L dNTPs (2 μl), 0.8 μl of each primer (5 μmol/L), FastPfu polymerase (0.4 μl), and template DNA (10 ng). Extraction of the PCR products was done on 2% agarose gel, purified using a AxyPrep DNA Gel Extraction Kit (Axygen Biosciences, Union City, CA, USA) and quantified using QuantiFluor™-ST (Promega, Madison, WI, USA) as described by the respective manufacturers. Amplicons (purified) were pooled in equimolar, followed by paired-end sequencing (2 × 300) on an Illumina MiSeq system (Illumina, San Diego, CA, USA).

### 2.5 Processing of Sequencing Data

This procedure was conducted at Majorbio Bio-Pharm Technology Co., Ltd. (Shanghai, China). Raw fastq files were demultiplexed, filtered by Trimmomatic for quality, followed by merging by FLASH as described previously (30, 31). Details related to the processing of sequencing data can be found in *eMethods* in ESM.

### 2.6 Untargeted Metabolomics Analyses

Fecal samples (50 mg) and serum samples (100 μl) were spiked with 40 μl of a methanol/water (4:1, *v*/*v*) solution (Merck, Darmstadt, Germany) and extracted as described by the manufacturer (Majorbio Bio-Pharm Technology Co., Ltd., Shanghai, China). The extracted samples were evaluated by liquid chromatography–mass spectrometry (LC-MS) (Shimadzu, Tokyo, Japan). Aliquots from all extracted samples were pooled to form a quality control (QC) sample, which was analyzed as done for the analytical samples (*eMethods* in ESM**)**.

### 2.7 Inflammatory Biomarker Assays

The concentrations of serum cytokines (CCL4, IFN-α, IFN-γ, IL-1β, IL-7, IFN-β, IL-1ra, IL-10, IL-4, IL-6, IL-8, IL-12 p40, IL-18, IL-17, and TNF-α) were analyzed employing xMAPtechnology (LXSAHM-20, R&D Systems Inc., Minneapolis, MN, USA; HGAMMAG-301K-03, Merck Millipore, Burlington, MA, USA). Detailed method can be found in *eMethods* in ESM.

### 2.8 Statistical Analysis

Data are shown as the mean ± SEM. Differences in the clinical features between groups were evaluated using *t*-tests, chi-square, and Mann–Whitney *U* test in SPSS version 20.0. Differences among groups were analyzed by one-way ANOVA with Tukey’s test or false discovery rate (FDR) correction. The cutoff for significance was *p* ≤ 0.05. Differential abundance of the microbial taxa and LC-MS-based metabolites were determined by negative binomial distribution and the Wald test (1.0 was the threshold for log fold change). Microbial taxa whose sequences were <1,000 and present in <20% of the whole cohort were filtered. Microbial and metabolite characteristics with FDR-adjusted *p* < 0.01 were considered significant. Effect sizes were assessed by comparisons of the fold changes. Identification of co-abundant metabolite clusters was done using “WGCNA21” in R, along with official tutorials (https://horvath.genetics.ucla.edu). Associations between specific metabolites, microbial, predicted pathways, and clinical variables in the NMDAR encephalitis patient subgroup were determined by correlation network analysis with a >1 threshold for the correlation coefficient (based on Spearman’s rank correlation) and FDR-adjusted *p* < 0.05 (using the Benjamini–Hochberg procedure). Finally, potential microbiota-dependent predictors of outcomes were assessed by age, gender, and the NEOS score, which is known to be a predictor of the 1-year functional status of NMDAR encephalitis patients (28, 32). We analyzed time to relapse with the Cox proportional hazards model, including the alpha diversity index as a factor, and provided the results as hazard ratios (HRs) with 95% confidence intervals (CIs).

## 3 Results

### 3.1 Demographic and Clinical Features of the Recruited Subjects

Fifty-eight patients with NMDAR encephalitis (mean age = 34.4 years, SEM = 1.8), among whom 36 patients had mRS scores of 2–3 (mean age = 35.3 years, SEM = 2.2), 22 patients had mRS scores of 4–5 (mean age = 33.1 years, SEM = 3.0), and 49 were HCs (mean age = 32.0 years, SEM = 1.5), were enrolled. The detailed demographics of the participants are shown in **Table 1**. The differences in age, female-to-male ratios, BMI, and nutritional factors among all of the groups were not significant. In addition, no obvious effect of sex and age on the gut microbiome structures was observed in this cohort (analysis of similarities: *p* > 0.05; **Supplementary Figure S1**).

**Table 1:** Extrinsic host factor profile including diet, stool consistency, and lifestyle in anti-*N*-methyl-d-aspartate receptor (NMDAR) encephalitis patients and control individuals.

	NMDAR Encephalitis	Control	*p*-value
No. of patients	58	49	
Sex (F/M)	36/22	31/18	0.9^b^
Age (years), mean (SEM)	34.4 (1.8)	32.0 (1.5)	0.4^c^
Initial presentation			
Psychogenic, *n* (%)	28 (48.3)	0	
Seizure, *n* (%)	15 (25.8)	0	
Other, *n* (%)	15 (25.8)	0	
MRI, abnormality, *n* (%)	30 (51.7)	0	
EEG, abnormality, *n* (%)	40 (68.9)	0	
CSF, abnormality, *n* (%)	29 (50)	0	
ICU admission, *n* (%)	14 (24.1)	0	
NEOS score, median (IQR)	2 (1–3)	0	
Gastrointestinal distress			0.4^b^
Frequently, *n* (%)	4 (6.8)	1 (2.0)	
Occasionally, *n* (%)	16 (27.5)	13 (26.5)	
None, *n* (%)	38 (65.5)	35 (71.4)	
Diarrhea (nearly 3 months)	7 (12.0)	5 (10.2)	0.7^b^
Defecation frequency			0.001^b^
3 times/day, *n* (%)	6 (10.3)	1 (2.0)	
1–2 times/day, *n* (%)	21 (36.2)	31 (42.8)	
Once/2 days, *n* (%)	15 (25.8)	17 (34.7)	
Once/3 days, *n* (%)	16 (27.6)	0 (0)	
Defecation consistency^a^			0.1^b^
Separate, hard lumps	2 (3.5)	0 (0)	
Lumpy, sausage-shaped	5 (8.6)	2 (4.1)	
Sausage-shaped with cracks	8 (13.8)	4 (8.1)	
Smooth and soft	32 (55.2)	28 (57.1)	
Soft with edges	9 (15.5)	15 (30.6)	
Mushy with ragged edges	2 (3.4)	0 (0)	
Liquid	0 (0)	0 (0)	
Dietary intake, median (IQR)			
Energy	1,827.2 (784.1–1988.2)	2,492.6 (802.4–2,691.1)	<0.001^d^
Protein	73.1 (57.7–99.3)	93.7 (69.4–125.7)	0.01^d^
Fat	71.2 (32.8–108.4)	82.2 (67.4–132.0)	0.05^d^
Fiber	18.5 (9.9–32.6)	23.9 (14.2–31.3)	0.2^d^
Carbohydrate	232.9 (158.1–294.6)	304.7 (219.4–479.5)	<0.001^d^
Cholesterol	128.3 (76.7–243.0)	164.6 (109.7–270.8)	0.5^d^
Retinol equivalent	818.3 (336.1–1,333.3)	1,053.0 (694.8–1,368.0)	0.5^d^
Vitamin B1 (mg)	1.3 (0.9–1.9)	1.5 (1.0–2.3)	0.3^d^
Vitamin B2 (mg)	1.4 (0.7–2.5)	1.3 (1.1–1.9)	0.6^d^
Vitamin B12 (μg)	0.44 (0.3–0.0)	0.34 (0.30–0.91)	0.2^d^
Folvite (μg)	450.8 (290.3–478.2)	473.9 (106.1–500.1)	0.7^d^
Vitamin E (mg)	36.0 (16.9–61.3)	49.6 (40.1–78.4)	0.1^d^
Vitamin C (mg)	166.1 (62.9–274.9)	211.9 (136.7–275.1)	0.5^d^
Na (mg)	3,284.8 (928.3–7250.7)	5,024.4 (3,585.5–7,265.2)	0.1^d^
Ca (mg)	672.9 (217.9–1290.2)	876.1 (620.5–1,135.0)	0.2^d^
Fe (mg)	28.0 (17.3–44.3)	35.1 (25.3–49.3)	0.08^d^
I (mg)	3.0 (1.9–3.4)	2.9 (0.7–3.2)	0.7^d^

Measurement data are expressed as the median (IQR). NEOS score, delayed treatment, improvement delay, abnormal CSF and MRI were strongly associated with the probability of poor functional status.

IQR, interquartile range; MRI, magnetic resonance imaging; EEG, electroencephalogram; CSF, cerebrospinal fluid; ICU, intensive care unit; NEOS score, anti-NMDAR encephalitis one-year functional status score

a Defecation consistency was assessed by type 4 Bristol stool scale.

b χ^2^ test.

c Student’s t-test.

d Mann–Whitney U test.

### 3.2 Fecal Microbiota Are Significantly Different in the Severe Disease Group

**Figure 2:**
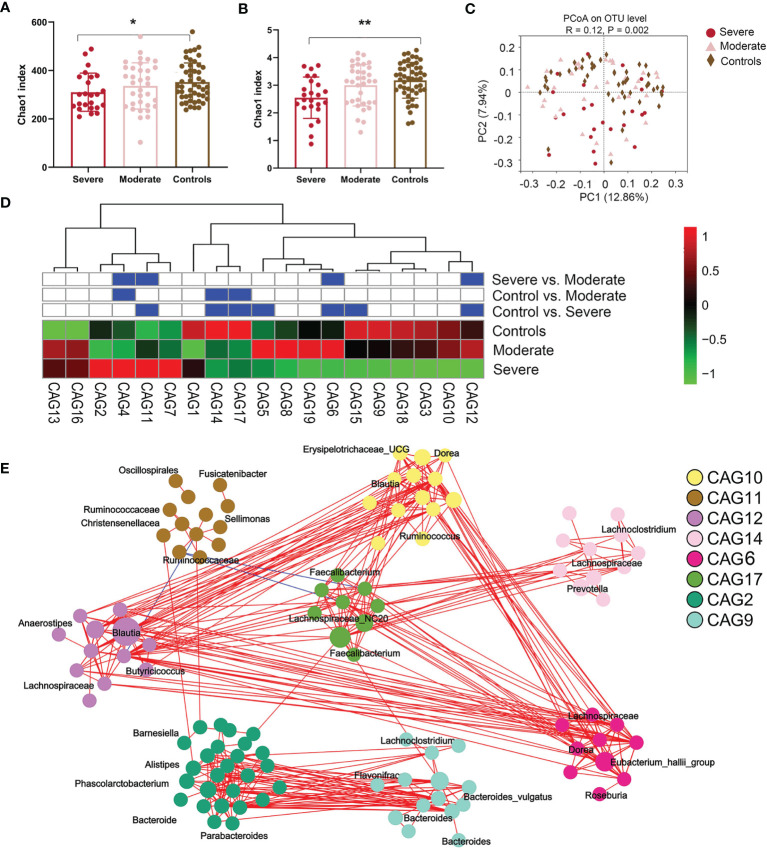
The fecal microbiota from anti-*N*-methyl-d-aspartate receptor (NMDAR) encephalitis patients with more severe status are distinct from those of healthy controls. **(A, B)** Comparison of the Chao 1 **(A)** and Shannon **(B)** indices between the different disease severity groups and healthy controls. A significantly decreased intestinal microbiota diversity in NMDAR encephalitis patients with more severe disease status was found. The mean values and standard deviations are presented as *bars*. *p < 0.05, **p < 0.01. *P-*values are from the Kruskal–Wallis test. **(C)** The overall bacterial signatures between the three groups were markedly different (unweighted Unifrac, PERMANOVA: *p* = 0.003). **(D)** Abundances of the 19 co-abundance groups (CAGs) in various NMDAR encephalitis severity subgroups. Abundances were transmuted into *Z* scores by subtracting the average abundances and dividing the standard deviations for all samples. *Z* scores were negative (shown in *green*) when row abundance was below the mean. CAGs at *p* < 0.05 (by Wilcoxon rank-sum test) are shown in *blue*. **(E)** Operational taxonomic unit (OTU)-level diagram for the enrichment of OTUs in various groups based on the markedly altered CAGs. *Node size* denotes the mean abundance of each OTU. Bacteria denoted on *nodes* were of the lowest classification status that could be identified by the RDP classifier. *Lines between nodes* denote associations, with *line width* showing association magnitudes, *red* denotes positive association, and *blue* denotes negative associations. *Lines* corresponding to associations with magnitudes >0.4 were drawn.

We sequenced fecal 16S rRNA genes in V3–V4 regions to evaluate variations in the gut microbiome in NMDAR encephalitis subgroups. Differences in the diversity of the gut microbiota between patients in the moderate status and HC groups were not significant. However, the severe group exhibited significantly lower values of the Chao1 (310 ± 78 vs. 345 ± 89, *p* = 0.04; **Figure 2A**) and Shannon (2.6 ± 0.8 vs. 3.1 ± 0.6, *p* = 0.006; **Figure 2B**) indices than those of HCs. Then, a score plot of the principal coordinate analysis (PCoA) based on unweighted UniFrac distances was built to evaluate the gut microbiota structure. The microbial structure and composition markedly varied between patients with different severity grades and HCs (*R* = 0.12, *p* = 0.002; **Figure 2C**).

Since bacteria form functional groups in the gut, we constructed a co-abundance network, whereby 294 operational taxonomic units (OTUs) were shared by ≥20% of samples based on SparCC correlation coefficients, and clustered the OTUs into 19 CAGs. Among them, CAG6 and CAG12 decreased and CAG11 increased markedly in patients in the group with more severe disease relative to patients with moderate status and to HCs (Wilcoxon rank-sum test: *p* < 0.05; **Figure 2D**). Among the OTUs in these CAGs depleted in the severe group, 80% belonged to *Bacteroides*, *Eubacterium_hallii_group*, *Anaerostipes*, *Megamonas*, *Ruminococcus*, *Butyricicoccus*, and *Faecalibacterium*, the members of which may alleviate inflammation. On the other hand, CAG11 comprised *Enterococcus*, *Oscillospirales*, *Fusicatenibacter*, and *Sellimonas*, which are opportunistic pathogens or pathogens (33) (**Figure 2E**).

**Figure 3:**
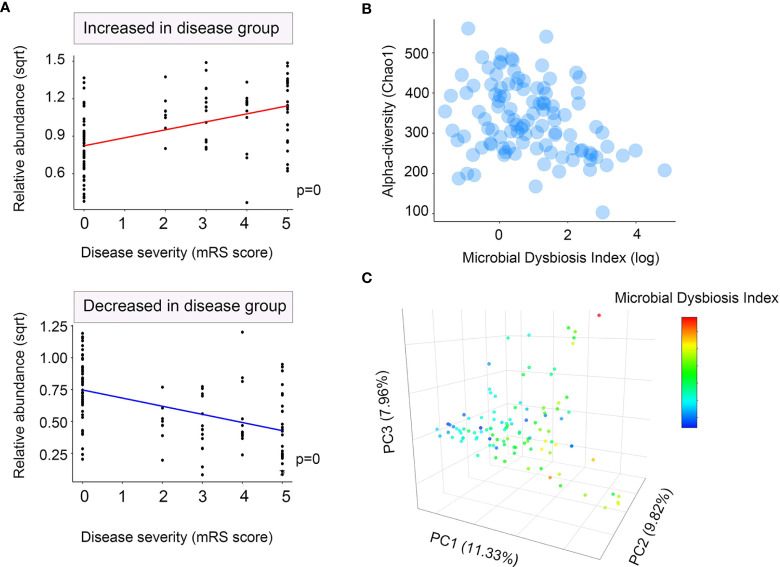
Microbial dysbiosis index (MD-index) characterizing anti-*N*-methyl-d-aspartate receptor (NMDAR) encephalitis severity. **(A)** Scatter plot of arcsine square root transmuted abundance for all summed abundances for the taxa decreased (*bottom*) or increased (*top*) in patients with NMDAR encephalitis versus the modified Rankin scale (mRS) score as a disease severity measure. **(B)** Scatter density plot for species richness vs. the MD-index for each sample. In samples with a high MD-index, a strong reduction in the species richness was observed. **(C)** Principal coordinate plot for unweighted UniFrac distance, colored using the MD-index. It can be noticed that the three principal coordinates stratified the samples by MD-index, which exhibited negative correlations with species richness. *Sqrt*, square root.

We then determined the log of [abundance in microbes increased in NMDAR encephalitis] over [abundance of microbes decreased in NMDAR encephalitis] for all samples, defined as the microbial dysbiosis index (MD-index) (34). The MD-index, which was disease phenotype-based, revealed strong positive correlations with disease severity (mRS score, *p* < 0.0001; **Figure 3A**) and negative associations with species richness (*p* < 0.0001; **Figure 3B**), implying that a severe disease status exhibits a markedly reduced species diversity, with a preference for extreme dysbiosis. Moreover, this index captured the overall beta diversity, resulting in a clear gradient by which samples grouped across all sample types (**Figure 3C**).

### 3.3 Metabonomics of the Fecal and Serum Specimens From NMDAR Encephalitis Patients Are Distinct From Those of Controls

All 107 participants were enrolled in the fecal metabonomic study, and a subset of 70 participants (39 patients with NMDAR encephalitis and 31 HCs) from this study was included in the serum metabonomic study. Identification of the differentially enriched metabolites that were significant was based on a variable importance in projection (VIP) threshold >1 and *p* < 0.05. Overall, 298 known fecal metabolites, 158 elevated and 140 suppressed, were identified in patients relative to HCs, which were visualized in a volcano plot (see **Supplementary Figure S2A**). The top priority metabolites identified were l-carnitine, samin, lysophosphatidic acid (lysoPA, a-25:0/0:0), and phenylalanyl-lysine, among others (see **Supplementary Figure S2B**). For the serum samples, 378 known metabolites, 58 elevated and 320 suppressed, separated patients with NMDAR encephalitis from normal controls (**Supplementary Figure S2C**). The top serum metabolites included 2-deoxy-d-gluconate, theophylline, and guanosine (**Supplementary Figure S2D**).

**Figure 4:**
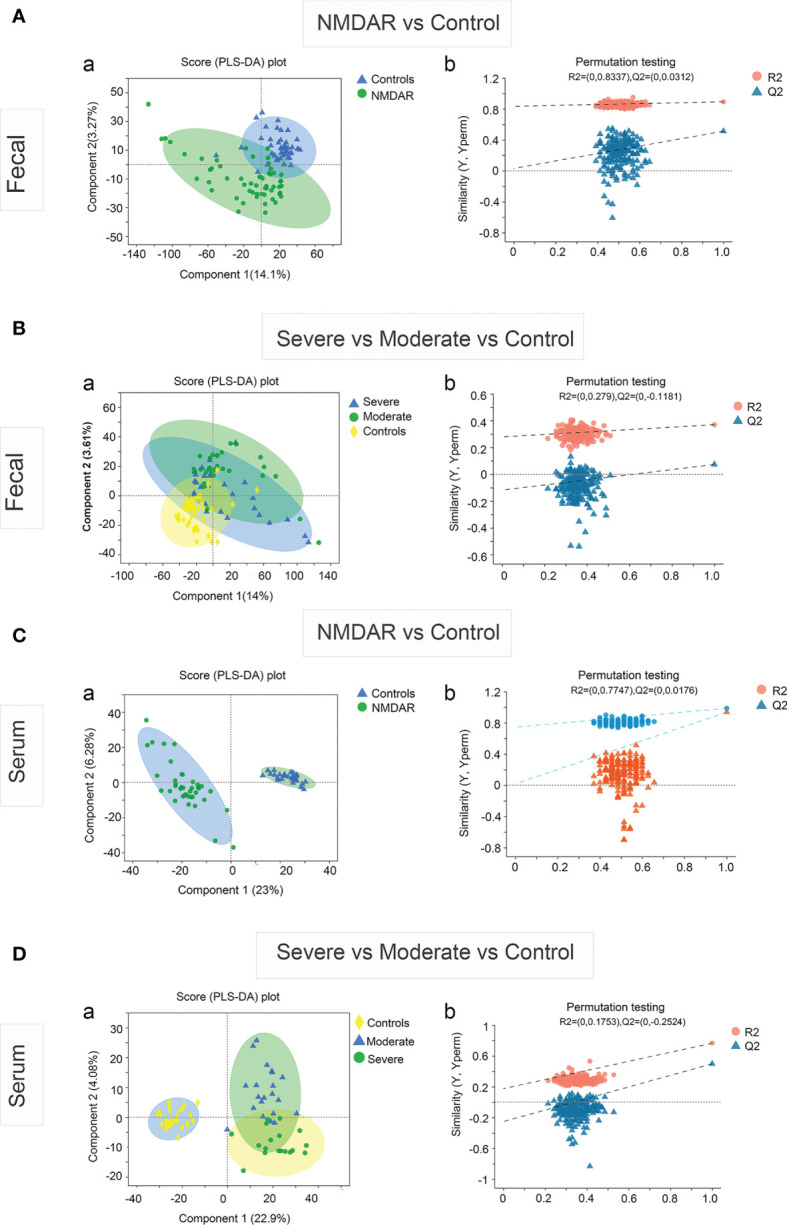
Partial least squares discriminant analysis (PLS-DA) of the fecal and serum metabolomes in anti-*N*-methyl-d-aspartate receptor (NMDAR) encephalitis cases versus healthy controls. Fecal **(A)** and serum **(C)** metabolomics PLS-DA score plot of NMDAR encephalitis cases (*blue*) vs. controls (*green*). Each *dot* denotes an individual subject. Permutation test for the PLS-DA model: 999 permutations led to intercepts of *R* ^2^ = 0.833, Q2 = 0.03 (fecal) and *R* ^2^ = 0.774, Q2 = 0.017 (serum), implying an acceptable model minus overfitting. Fecal **(B)** and serum **(D)** metabolomics PLS-DA score plot of NMDAR encephalitis cases with severe status (*blue*) and moderate status (*green*) versus controls (*yellow*). Each *dot* represents an individual subject. PLS-DA model permutation test: 999 permutations led to intercepts of *R* ^2^ = 0.279, Q2 = −0.118 and *R* ^2^ = 0.175, Q2 = −0.252, implying an acceptable model minus overfitting.

The partial least squares discriminant analysis (PLS-DA) plot showed the global fecal metabolic changes in NMDAR encephalitis patients and HCs (**Figure 4A**). A separation between patients with different disease severity status and HCs was also obtained with the PLS-DA method (**Figure 4B**). The serum data recapitulated the distinction, classifying the patients into different severity grading and HC groups (**Figures 4C, D**). However, the Q2 values (**Figure 4B**) for all the comparisons were low or negative, which means that the statistical model may not be reliable. Thus, in this study, NMDAR encephalitis patients did not show significantly different metabolite profiles relative to HCs. In addition, the metabolite levels did not shift with disease severity.

**Figure 5:**
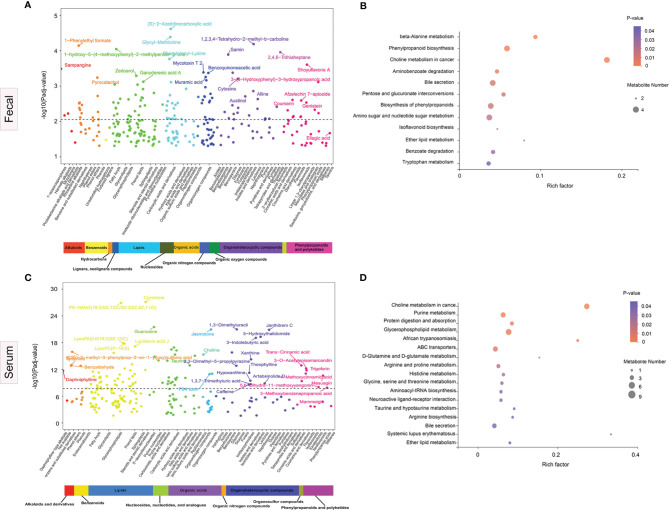
Significance plot for the adjusted selected differential metabolites in anti-*N*-methyl-d-aspartate receptor (NMDAR) encephalitis cases vs. healthy controls by sub- and super-pathway level. **(A, C)** Each significantly selected fecal **(A)** and serum **(C)** metabolites sub-pathway is represented. The −log10(*P*_adj_) for each metabolite is displayed by respective sub- and super-pathway (*P*_adj_, adjusted *p*-value). Unadjusted Wilcoxon rank-sum test *p*-values were adjusted for multiple comparisons using Benjamini–Hochberg (BH) correction to yield adjusted *p*-values. The *dashed line* represents the adjusted *p*-value. *Asterisks* represent compounds that were not verified against a standard, but whose identity the analytical platform was confident in. **(B, D)** Significantly enriched sub-pathways from metabolites selected using partial least squares discriminant analysis (PLS-DA) models are illustrated in dot plots. Each significantly selected fecal **(B)** and serum **(D)** sub-pathway is denoted by a *circle* described by three parameters. *Circle size* shows how many of the metabolites were selected in the sub-pathway (see legend in *gray* to the *right of the plot* for relative sizes). *Circle shades* from *light pink* to *red* denote selected sub-pathway significance levels based on −log10(*p*-value) (see *legend to the right of the plot* for relative color gradient). Sub-pathways were markedly enriched if *p* < 0.05, which was comparable to −log10(*p*-value) > 1.3. *Circle position along the rich factor axis* shows the abundance of the selected metabolites from the sub-pathway against all sub-pathway metabolites.

Sub-pathways shared by metabolites provide meaningful groups and are vital for elucidation of disease mechanisms. In multivariate models controlling for three potential confounders, namely, age, sex, and BMI, we identified 277 differential fecal metabolites across 51 sub-pathways and 12 super-pathways. Similarly, for serum, we identified 221 differential metabolites across 47 sub-pathways and 9 super-pathways. **Figures 5A, B** illustrate the fecal and serum metabolites with the highest variations in abundance between the disease cases and HCs, respectively.

Fecal metabolomics pathway enrichment assessments revealed significantly overrepresented sub-pathways among the differentially abundant metabolites. PLS-DA revealed 17 sub-pathways, including, among the most significant, beta-alanine metabolism, phenylpropanoid biosynthesis, choline metabolism in cancer, aminobenzoate degradation, bile secretion, pentose and glucuronate interconversions, and tryptophan metabolism, as represented by the dot plot (**Figure 5C**). For serum pathway enrichment analysis, PLS-DA identified 13 sub-pathways that were significantly different between disease cases and HCs, including choline metabolism in cancer, d-glutamine and d-glutamate metabolism, purine metabolism, glycerophospholipid metabolism, ABC transporters, and arginine and proline metabolism (**Figure 5D**).

The enrichment analysis suggested that the choline metabolism in cancer pathway and the bile secretion pathway were altered significantly in both serum and fecal samples in patients with NMDAR encephalitis, indicating that these two metabolic pathways are important in NMDAR encephalitis.

### 3.4 The Differential Genera Showed a Correlation with Dysregulated Cytokines in NMDAR Encephalitis

**Figure 6:**
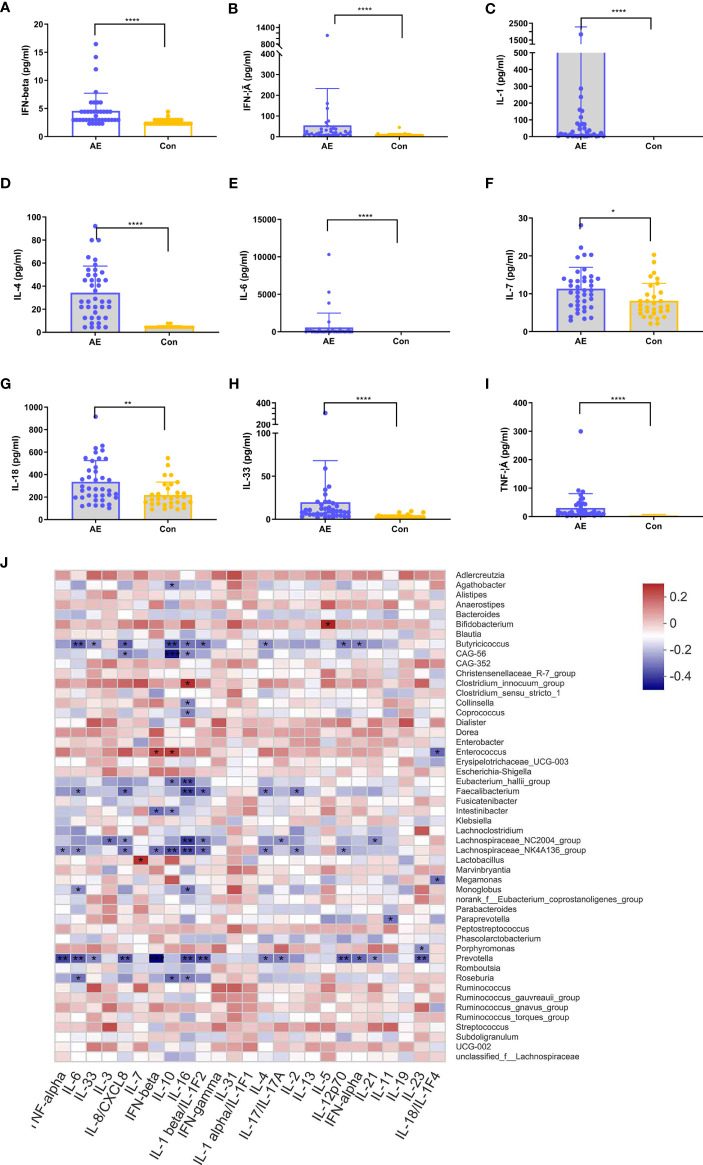
Correlation between dysregulated cytokines and differential genera in anti-*N*-methyl-d-aspartate receptor (NMDAR) encephalitis. **(A–I)** Analysis of 12 immune cytokines in patients with NMDAR encephalitis and in healthy controls (HCs). Participants’ sera were run on Luminex. Differences are determined by a *t*-test. **(J)** Correlation analyses of cytokines and differentially enriched genera between any two groups. Abundance rates of differentially enriched species (top 30) in HCs and NMDAR encephalitis were evaluated for association with differential immune factors using Spearman’s correlation analysis. Correlations are shown by color gradients from *green* (negatively correlated) to *purple* (positively correlated). Correlation coefficients and *p*-values (*p < 0.05, **p < 0.01, ***p < 0.001, **** *p* < 0.0001) are shown.

In various immune-associated disorders, cytokines are key drivers of inflammation and tissue damage (35). Commensal microbiome modulates cytokine-mediated immune reactions (36, 37). To establish the relationship between cytokines and the gut microbiota in NMDAR encephalitis patients, we evaluated the serum levels of inflammatory cytokines in NMDAR encephalitis patients relative to HCs (**Figures 6A–I**). Nine cytokines exhibited significantly higher levels in the patient group than in HCs, including IFN-β, IFN-γ, IL-3, IL-6, IL-1, IL-7, IL-18, IL-33, and TNF-α. These cytokines were elevated in patients with severe status and were suppressed in HCs, while a small increase in patients with moderate status further verified that cytokines are associated with disease severity grading (data not shown).

To identify the immune disorder-associated bacteria in NMDAR encephalitis patients, correlation analyses of the various genera and different cytokines between any two groups were performed. **Figure 6J** shows that *Bifidobacterium*, *Enterococcus*, *Clostridium_innocuum_group*, and *Roseburia*, which were the significantly increased genera in the severe disease status group relative to HCs, were positively correlated with the levels of cytokines, which revealed these bacterial genera to be the core bacteria involved in the stimulation of these immune factors. In contrast, *Butyricicoccus*, *Eubacterium_hallii_group*, *Lachnospiraceae_NC2004_group*, *Faecalibacterium*, *Lachnospiraceae_NK4A136_group*, and *Prevotella*, which were significantly reduced in severe NMDAR encephalitis patients, showed a strong negative association with immune factors, implying that these genera could reduce the inflammatory cytokine levels in NMDAR encephalitis patients. However, there was a relatively small marked positive association between the different genera and immune factors in HCs and moderate groups.

### 3.5 Association Between Metabolites, Cytokines, and Gut Microbiota in NMDAR Encephalitis

At an FDR of 5%, five gut microbiota CAGs were markedly associated with 8 serum metabolic modules, while 7 gut microbiota CAGs were markedly associated with 8 fecal metabolic modules. These metabolic modules were also respectively associated with serum cytokines.

**Figure 7:**
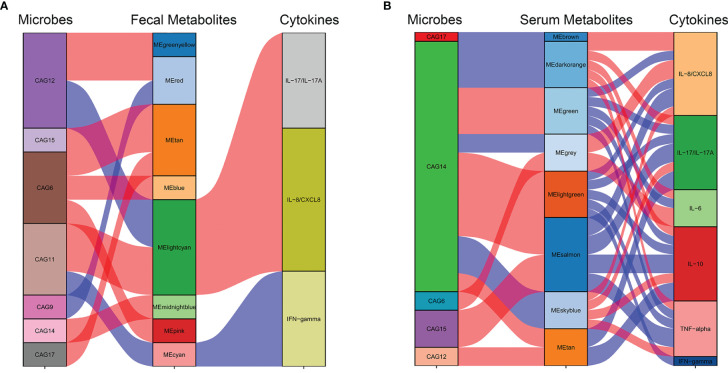
Interrelationships between the gut microbiota composition, host metabolic profiles, and cytokine phenotypes. Correlation network between the gut microbiota of significant co-abundance groups (CAGs), fecal **(A)** and serum **(B)** metabolites, and cytokines. *Red lines* denote positive correlations (FDR < 0.05), while *blue lines* denote negative correlations (FDR < 0.05). In the gut microbiota column, *green* stratum denotes CAGs highly enriched in the control group. Stratum in *brown* denotes microbiota increased in the more severe group. In the metabolomics column, *orange* and red *strata* denote anti-*N*-methyl-d-aspartate receptor (NMDAR) encephalitis-negative metabotypes, while *blue* and *green* strata denote NMDAR encephalitis-positive metabotypes.

The correlation between CAGs, fecal metabolic module, and cytokines can be seen in **Figure 7A**. The light cyan metabolic module (characterized by prenol lipids, steroids, and steroid derivatives) enriched in NMDAR encephalitis, was positively correlated with CAG11 (more abundant in the severe disease group) and negatively correlated with CAG12 (enriched in HCs). CAG11 also showed a negative association with the cyan module enriched in HCs (characterized by glycerophospholipids and fatty acyls; *p*-value for interaction, <0.05).

The correlation between CAGs, serum metabolic module, and cytokines can be seen in **Figure 7B**. CAG6, enriched in the NMDAR encephalitis patients, was positively correlated with the serum metabolic modules that were “NMDAR encephalitis-positive associated”, such as the gray module (enriched in organic acids and derivatives and fatty acids; *p*-value for interaction, <0.05). On the other hand, CAG14, CAG17, and CAG15, enriched in the HC group, were negatively associated with metabolic modules that were enriched in the disease group, including the brown, dark orange module (characterized by glycerophosphocholines and glycerophosphoethanolamine-related metabolites; *p*-value for interaction, <0.05), sky blue module (characterized by lipid-like molecules, organic acids and derivatives, and amino acids; *p*-value for interaction, <0.05), and the gray module (characterized by organic acids and derivatives; *p*-value for interaction, <0.05). Particularly, CAG14, mainly consisting of Lachnospiraceae and *Prevotella*, was closely associated with 7 serum modules, implying that it might have vital functions in the maintenance of normal physiology by interacting with various serum metabolites.

Based on the marked associations between distinctive metabolic characteristics in fecal and serum samples and the disorderly gut microbiota in NMDAR encephalitis, it is possible that gut microbiota dysbiosis initiated the disordered microbial functions, leading to the deficiency of anti-inflammation protective metabolites and dysregulation of cytokines, thereby increasing vulnerability to NMDAR encephalitis.

### 3.6 Fecal Microbiota and Metabolites Are Predictive of Clinical Outcomes in NMDAR Encephalitis Patients

Outcomes were evaluated in all 58 patients: 21 (36.2%) patients had an unfavorable outcome at 6 months, including 1 (1.7%) who died within 3 months after admission. Then, we determined whether the key characteristics of the fecal microbiome (bacterial diversity and community composition) or metabolites (fecal and serum) are predictive of the clinical outcomes of NMDAR encephalitis patients. The mRS score, which was the main outcome, was measured at 6 months post-admission. The secondary outcome was clinical relapse.

**Figure 8:**
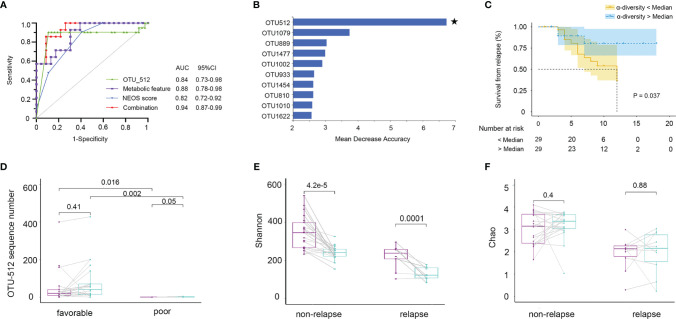
Fecal microbiota and outcomes in anti-*N*-methyl-d-aspartate receptor (NMDAR) encephalitis patients. The community composition of fecal bacteria was significantly predictive of functional outcomes at 6 months. **(A)** Diagnostic outcomes are presented *via* receiver operating characteristic (ROC) curves for the outcomes at 6 months of NMDAR encephalitis patients. **(B)** Detailed explanatory variables that are based on random forest models for comparison of favorable and worse outcomes. *Bar lengths* in the histogram denote the mean decrease accuracy, showing the significance of operational taxonomic units (OTUs) for classification. Random forest identified the fecal-associated OTU512 (*Granulicatella*) as the strongest predictor of favorable outcome. **(C)** Cox proportional hazards model of the association of relapse. Patients were grouped into two: α-diversity < median and α-diversity > median, for which at least 6-month Kaplan–Meier survival plot is shown. Fifty-eight NMDAR encephalitis patients were grouped based on Shannon index median (log-rank *p* = 0.037, HR = 2.74, 95%CI = 1.06–7.05). Numbers below the curve represent the at-risk patients per group. *Yellow line*, below the median; *blue line*, above the median. Analyses were done using quantitative values, and grouping by quantiles was used for graphical presentation only. Patients with unfavorable outcomes did not improve microbiota abnormalities compared to patients with favorable outcomes. **(D)** Patients with paired baseline and long-term (6 ± 1 months) fecal samples are represented (*n* = 30). The measured parameters were genera OTU512. **(E, F)** Patients without relapse had an overall improvement of the Shannon and Chao indices over time compared to patients with one or several relapses. Wilcoxon signed-rank test for comparisons of paired baseline and long-term change.

Firstly, we evaluated whether the community composition of fecal bacteria can predict NMDAR encephalitis outcomes. By using a random forest to assess poor outcome-associated taxa, we identified 10 OUTs most strongly predictive of higher mRS scores at 6 months after admission. The value of the area under the receiver operating characteristic curve (AUC) was 0.84 (the sensitivity and specificity were 72% and 86%, respectively) (**Figure 8A**). Then, we evaluated whether these taxa can predict long-term functional outcomes. **Figure 8B** shows that OTU512 (*Granulicatella*) was highly predictive of favorable disease outcomes. The association between the other OTUs detected and disease outcomes was not significant (*p* > 0.05). Logistic multivariate regression analysis indicated that a high abundance of OTU512 was correlated with a reduced risk of developing worse outcomes, independent of age, sex, disturbance of consciousness, and the NEOS score [odds ratio (OR) = 0.11, 95%CI = 0.02–0.5, multivariate *p* = 0.007]. The combination of clinical features (NEOS score), microbial marker (OUT-512), and a panel of metabolic markers in serum (kynurenine and choline) and feces (lysoPA and l-carnitine) could further enhance the performance of this discrimination significantly, which yielded an AUC of 0.94 (95%CI = 0.7–0.9) (**Figure 8A**).

We next investigated whether the diversity of fecal bacteria predicts NMDAR encephalitis outcomes. Bacterial diversity (as evaluated by the Shannon diversity index) was not a significant predictor of worse outcomes in NMDAR encephalitis (*p* = 0.9). Then, survival analysis showed the relationship between further relapse and microbial diversity (**Figure 8C**). In the low-bacterial-diversity (α-diversity < median, *n* = 29) group, the risk of patients developing relapse was significantly higher than that in the high-diversity (α-diversity > median, *n* = 29) group (log-rank *p* = 0.04, HR = 2.7, 95%CI = 1.0–7.0).

Subsequently, after at least 6 months of follow-up, we collected fecal sample from 30 NMDAR encephalitis patients of the same cohort (21 patients with favorable outcomes and 9 patients with poor outcomes in the recovery phase). We explored the longitudinal changes in dysbiosis and further validated the relationship between disease outcome markers and dysbiosis. The results showed that patients with worse outcomes had a significantly lower OTU512 than patients with favorable outcomes (**Figure 8D**). Moreover, patients with higher microbial diversity did not have relapse during the follow-up (**Figures 8E, F**).

We thus concluded that, in NMDAR encephalitis patients, poor clinical outcomes and relapse are both predicted by metabolites, community composition, and microbial diversity.

## 4 Discussion

This is the first multi-omics-based study to comprehensively evaluate the associations of the gut microbiome with fecal and serum metabolites and inflammatory cytokines from a large and prospective cohort including 107 individuals. This is also the first study to simultaneously show that differences in the fecal microbiota are a predictor of clinical outcomes in NMDAR encephalitis patients. The core findings of this study were as follows: i) both the gut microbiota and circulation metabolites markedly changed in patients with more severe disease than in patients with moderate status and HCs, which indicates that dysbiosis could increase the risk of susceptibility to severe disease; ii) two key features of the fecal microbiome at admission—enrichment of OTU512 and higher bacterial diversity—predicted favorable long-term outcomes and reduced further relapse in patients with NMDAR encephalitis; iii) the strong correlations between the distinguishing metabolic features, disordered gut microbiota, and cytokines in NMDAR encephalitis suggested that the fecal microbiome might play vital roles in the maintenance of normal physiological conditions by interacting with several metabolites and pro-inflammatory factors.

The results of the microbial features obtained in this investigation were largely consistent with those previously reported by Herken et al. (38), Xu et al. (39), and Chen et al. (21), namely, an increase in *Oscillospirales* and *Enterococcus* and a decrease in various organic acid-producing bacteria such as Lachnospiraceae, *Ruminococcus*, *Faecalibacterium*, *Anaerostipes*, and *Prevotella*. We further confirmed that these taxa were altered significantly in patients with more severe disease compared with those of moderate status and the HCs. Notably, we also found some taxa depleted in NMDAR encephalitis that were not observed in previous studies, including *Eubacterium_hallii_group*, *Megamonas*, and *Butyricicoccus*. In contrast, Herken et al. described that both patients and controls had a normal gut microbiome (38). In the study of Chen et al. (21), *Bacteroides* were shown to be higher in NMDAR patients, yielding opposite results in comparison with our study. Previous reviews suggested that *Bacteroides* plays an important role in polysaccharide metabolism and host immune regulation (40, 41). This lack of reproducibility among studies probably resulted from several factors including population heterogeneity, sequencing methods and depths (raw reads per sample ranging ~40K in the previous V4 region of bacterial 16S rRNA gene sequencing studies compared to ~110K in this study), small sample size, different disease stages, a less rigorous inclusion criteria (included 3 ovarian teratoma patients with an identified trigger), and inadequate control of important confounding factors, particularly immunotherapy, antibiotic treatment, and diet.

Furthermore, ecologically, gut bacteria exist as functional groups referred to as “guilds” (42). In response to changes in physiological and environmental resources, key members of co-abundance groups could thrive or decline together (43). Thus, relative to the conventional taxon-based analysis, CAG-based analysis provides a more ecologically relevant method for facilitating the identification of functionally vital gut microbiota in NMDAR encephalitis. In our study, the abundance of CAG11 significantly increased in patients with severe status. This CAG contained several pathogens or opportunistic pathogenic bacteria, such as *Enterococcus*, Oscillospirales, *Fusicatenibacter*, and *Sellimonas*. Thus, these bacteria may initiate innate immune responses through lipopolysaccharide production and elicit successive inflammatory responses that are a result of local cytokine generation.

Our study also provided the important information that a reduced fecal bacterial diversity, including the Shannon and Chao1 indices, predicts further relapse and correlates positively with disease status (mRS score). The mRS score is an important informative parameter for disease severity in numerous neurological diseases (44). A low alpha diversity is a dysbiosis marker and denotes “worse” health (45–47). By using random forest analysis, the combination of microbial taxa, metabolic makers, and the NEOS score at admission can even distinguish patients with adequate outcomes from those with worse outcomes, with an AUC of 0.94. This result was validated by the longitudinal microbiota analysis. Thus, combination with fecal microbiome could also be useful in the early identification of patients with poor prognostic outcomes and inform whether early second-line immunotherapy or other novel salvage treatments can be administered to NMDAR encephalitis patients.

Metabolomes reflect the cumulative effects of endogenous physiological processes and exogenous factors (48). The human gut microbiome comprehensively interacts with the host *via* substrate co-metabolism and metabolic exchange. PLS-DA, highly used for the identification of metabolites that markedly separate controls from cases, identified l-carnitine and lysoPA as differential fecal metabolites in NMDAR encephalitis patients versus controls. l-Carnitine is an amino acid derivative that participates in the transport of long-chain fatty acids into the mitochondria. It improves mitochondrial functions and exerts beneficial effects in neurological disorders (49). An upregulated lysoPA correlated with the occurrence of several inflammatory conditions, such as asthma (50), cancer (51), and autoimmune hepatitis (52). We identified phosphatidylserine (PS), phosphatidylethanolamine (PE), and lysoPA as differential serum metabolites in NMDAR encephalitis patients versus controls. According to most previous studies, these glycerolipids (glycerophosphocholines and glycerophosphoethanolamines) are the major pro-inflammatory glycerolipid derivatives that are absorbed into the bloodstream (53, 54). Phosphocholines, which are low-density lipoprotein (LDL, “bad”) cholesterol components, interact with C-reactive proteins in a pro-inflammatory and pro-atherosclerotic manner (55–57).

The pathway enrichment analysis in our study suggested that the choline metabolism pathway and the bile secretion pathway were significantly altered in both serum and fecal samples in patients with NMDAR encephalitis. Several studies have shown that choline metabolism plays pathophysiological roles in central nervous system (CNS) diseases. For instance, choline metabolism has a potentially prognostic role in cognitive impairment after stoke (58). Choline is the precursor of neurotransmitter acetylcholine and also acts as an agonist for the receptors that could regulate CNS immune responses, and their dysregulation is associated with the pathogenesis of several neurological diseases (59).

The main strengths of this study were as follows: i) the gut microbiota in cohabiting spouses exhibit high similarities relative to non-cohabiting age-matched male–female pairs; thus, recruiting paired spousal and sibling controls could largely minimize potential confounding. ii) Although some studies have highlighted the inconsistent sex and age influences on the gut microbiome, no obvious effects of sex and age on the gut microbiome structures were observed in this cohort based on beta diversity (**Supplementary Figure S2**). iii) The paired fecal and serum samples of the same cohort may provide more insights that deviations in gut microbial communities are associated with disease severity *via* mediation of fecal and serum metabolites. iv) The relatively large sample size and prospective follow-up data, along with longitudinal microbiome analysis, enabled us to better understand the specific bacteria that may contribute to susceptibility, worse prognosis, and relapse.

We also acknowledge various limitations of this study. Firstly, participants were recruited from Western China; thus, the reported results might not be applicable to Western populations or other populations with specific genetic backgrounds and diets. Secondly, in our study, drugs including immunotherapies and antibiotic were not used on all patients, but other drug use was inevitable since most NMDAR encephalitis require anti-seizure (diazepams, phenobarbital, or midazolam) or anti-antipsychotic drugs at initial onset during emergency care. Hence, although we have obtained the samples from patients as soon as possible, so that the impacts of the above drugs have been reduced to a very low level, the potential impacts of the above drugs on the gut microbiota and metabolites cannot be completely excluded. In addition, similar to other human studies, even though our results were significant when controlled for crucial confounders, we did not control for all potential clinical exposures. For example, dietary data were obtained using food questionnaires, and inaccuracies of self-reported data can weaken the power in dietary analyses. Furthermore, NMDAR encephalitis patients at disease onset had higher constipation or diarrhea rate in comparison with healthy controls (38% vs. 2%) since autonomic dysfunction is a common symptom in patients with NMDAR encephalitis. A previous study also reported that the incidences of constipation account for nearly one-third of NMDAR encephalitis patients. However, differences between stool frequencies would make it difficult to explain the causal relationship between stool characteristics and microbiota alterations. Finally, the LC-MS platform has certain limitations, e.g., metabolites need to be ionized in order to be detected, and it tends to be highly susceptible to variability. Thus, the combination of MS and NMR methods could be used for complementary sets of metabolites, thereby allowing for a comprehensive assessment of the metabolome. In conclusion, despite our findings highlighting the clinical relevance of the gut microbiome and metabolite changes in NMDAR encephalitis, various knowledge gaps remain. Fecal microbiota may not provide or reflect the whole bowel microbial environment; thus, mechanistic experimental models, and independent validation cohort, and human interventional studies targeting precise metabolic/microbial features should be performed to unravel how they contribute to various physiological roles and pathological outcomes.

## Data Availability Statement

The datasets presented in this study can be found in online repositories. The names of the repository/repositories and accession number(s) can be found below: https://www.ncbi.nlm.nih.gov/, BioProject: PRJNA764676.

## Ethics Statement

The studies involving human participants were reviewed and approved by the Institutional Review Boards of the Ethics Committee of West China Hospital of Sichuan University. The patients/participants provided written informed consent to participate in this study.

## Author Contributions

XG drafted and revised the manuscript, carried out the statistical analysis, and interpreted the data. YL, XL, AL, and KG collected the samples. DZ and ZH conceptualized and designed the study and revised the manuscript. All authors contributed to the article and approved the submitted version.

## Funding

This study is supported by the National Science Foundation of China (grants 81671291, 81420108014, and 81971213) and the National Key R&D Program of China (2018YFC1312300).

## Conflict of Interest

The authors declare that the research was conducted in the absence of any commercial or financial relationships that could be construed as a potential conflict of interest.

## Publisher’s Note

All claims expressed in this article are solely those of the authors and do not necessarily represent those of their affiliated organizations, or those of the publisher, the editors and the reviewers. Any product that may be evaluated in this article, or claim that may be made by its manufacturer, is not guaranteed or endorsed by the publisher.
