# Supplementary material for: Disturbance of Gut Bacteria and Metabolites Are Associated with Disease Severity and Predict Outcome of NMDAR Encephalitis: A Prospective Case–Control Study
Source: Front Immunol. 2022 Jan 3;12:791780. doi: 10.3389/fimmu.2021.791780 (PMC8761854; doi:10.3389/fimmu.2021.791780)
Supplement: Supplementary file 1 [file DataSheet_1.docx]

Supplementary Material

**Disturbance of Gut Bacteria and Metabolites Are Associated with Disease Severity and Predict Outcome of NMDAR encephalitis: A Prospective Case–Control Study**

Xue Gong, MD; Yue Liu, MD; Xu Liu, MD, PhD; Aiqing Li, MD; Kundian Guo, MD; Dong Zhou, MD, PhD; Zhen Hong, MD, PhD

**I eMethod**

1. **Extended Participant Information:**
   1. **Inclusion criteria**

We included patients who met the criteria for definite NMDAR encephalitis according to definitions from a recent consensus statement: (1) acute onset of 1 or more of the following 8 major manifestations: psychosis, memory deficit, speech disturbance, seizures, movement disorder, a disturbance of consciousness, autonomic dysfunction, and central hypoventilation within 3 months; (2) CSF tests positive for NMDAR antibodies (cell-based assay); and (3) reasonable exclusion of other disorders.

- 1. **Data collection**

We designed the data collection form, which includes the data of demographic, clinical, treatment, laboratory data and prognosis data were retrieved from the database of the One-WC (Outcomes of anti-NMDAR Encephalitis Study in Western China) registry study(1-3). The consistency and accuracy of the reported data were systematically assessed and collected by 2 neurologists.

The standardized data collection included: The standardized data collection included (1) demographic characteristics such as sex and age at disease onset; (2) clinical symptoms at onset including abnormal psychiatric, cognitive dysfunction, seizures, memory deficit, speech disturbance, movement disorder, disturbance of consciousness, autonomic dysfunction, and central hypoventilation; (3) comorbidities; (4) laboratory and radiographic findings (detailed determination of CSF and MRI examinations can be seen in the eMethods in the Supplement); (4) treatments; (5) time from disease onset to immunotherapy initiation; (6) clinical course during hospitalization; (7) mRS score at the initial onset; (8) mRS score after 1, 3, 6 and 12 months after admission. The standardized outcome data during follow-up was evaluated at every 2-3 months or when patients needed to report clinical worsening and relapse by clinic visit, which was objectively assessed by the treating neurologist.

In face-to-face questionnaire interviews, we collected host metadata, including data regarding gastrointestinal distress, defecation, dietary intake and Bristol stool score. A meal-based food frequency questionnaire (FFQ) was used to calculate baseline nutrient intake based on [China Food Composition (Book 1, 2nd Edition)]. Then, we used nutritional database (http://db.foodmate.net/yingyangsu/) to provide information regarding nutrients including carbohydrate, protein, fat, etc. All information provided during the interviews was recorded in the case report form.

- 1. **Evaluation prognosis**

Clinical functional outcomes were assessed through the modified Rankin Scale (mRS),19 which was used to classify patients who were able to walk without aid (mRS ≤2) or needed assistance while walking (mRS ≥3) (0 = asymptomatic; 1 = nondisabling symptoms that do not interfere with lifestyle; 2 = minor disabling symptoms that lead to some restriction of lifestyle but do not prevent totally independent existence; 3 = symptoms significantly interfering with lifestyle or preventing totally independent existence; 4 = moderately severe disabling symptoms that clearly prevent independent existence with total support needed for basic daily activities; 5 = severe disabling symptoms, totally dependent and requiring constant attention day and night; and 6 = death due to neurologic symptoms).

- 1. **Determination of antibodies to NMDAR and Clinical examinations**

CSF examinations of patients were performed within 1 week of disease onset, and determination of antibodies against NMDAR was performed using indirect immunofluorescence (IIF) as previously reported(1, 4). Patient serum and CSF samples were obtained simultaneously and were maintained and transferred on ice to the laboratory. All specimens (serum and CSF) were evaluated for anti-NMDAR IgG antibodies by indirect immunofluorescence (IIF) using EU 90 cells transfected with the The NMDAR1 subunit (NR1) of the NMDAR complex and immobilized on BIOCHIPs (euroimmun AG, Germany) as previously described(5). Slides were incubated with undiluted CSF samples or serum samples at a starting dilution of 1:10, and analysis was performed according to the manufacturer’s guidelines. Following incubation of samples with transfected or untransfected cell lines, slides were washed and stained with fluorescein-labeled anti-human IgG antibodies and visualized using a fluorescence microscope. Samples were classified as positive or negative based on the intensity of surface immunofluorescence of transfected cells compared to non-transfected cells, according to the manufacturer’s suggested recommendations for reading and interpretation.

Intracranial pressure was evaluated by a cerebrospinal fluid pressure gauge, and a pressure > 180 mmH2O was considered to be increased. Integrated CSF analyses included total cell count, total protein content, albumin, and IgG content in both CSF and serum. Abnormally elevated cell counts were defined as total white cell counts > 5/ml and CSF protein > 500 mg/L. The detection of organism-specific nucleic acids in CSF by polymerase chain reaction (PCR) was used for rapid diagnosis of central nervous system (CNS) infections, such as nucleic acid testing for herpes simplex virus (HSV), varicella zoster virus (VZV), and enteroviruses. In addition, cultures for bacteria, tuberculosis, and fungal infections of the CSF were all performed.

MRI data were obtained by experienced technician within 1 week of disease onset and at different timepoints during the course of the disease. MRI studies were performed using a Germany Siemens-Trio Erlangen 3.0 T MRI (12-channel coil). Regular MRI series including axial T2-weighted image (T2WI), T1-weighted image (T1WI), and fluid-attenuated inversion recovery image (Flair). Contrast-enhanced studies were obtained using intravenous gadopentetate dimeglumine. Extensive investigations for tumours were done, which included thoraco-abdominal CT, pelvic CT scan, positron emission tomography (PET), or transvaginal ultrasound.

1. **Microbiota Sequencing**
   1. **DNA Extraction and PCR Ampliﬁcation**

Microbial community genomic DNA was extracted from fecal samples using an E.Z.N.A.R Soil DNA Kit (Omega Biotek, Norcross, GA, United States) according to the manufacturer’s instructions. The DNA extract was checked on a 1% agarose gel, and the DNA concentration and purity were determined with a NanoDrop 2000 UV-Vis spectrophotometer (Thermo Fisher Scientiﬁc, Wilmington, UnitedStates). The V3-V4 hypervariable region of the bacterial 16S rRNA gene was ampliﬁed with the primer pair 338F (50-ACTCCTACGGGAGGCAGCAG-30) and 806R (50-GGACTACHVGGGTWTCTAAT-30) by an ABI GeneAmpR 9700 PCR thermocycler (ABI, CA, United States), with an eight-base sequence barcode unique to each sample at the 5’ end of 338F and 806R(6). PCR ampliﬁcation of the 16S rRNA gene was performed as follows: initial denaturation at 95◦C for 3 min, followed by 27 cycles of denaturation at 95◦C for 30 s, annealing at 55◦C for 30 s and extension at 72◦C for 45 s; a single extension at 72◦C for 10 min; and a hold at 4◦C. The PCR mixtures contained 5 × TransStart FastPfu buﬀer (4 µL), 2.5 mM dNTPs (2 µL), 5 µM forward primer (0.8 µL), 5 µM reverse primer (0.8 µL), TransStart FastPfu DNA Polymerase (0.4 µL), template DNA (10 ng), and ddH2O (up to 20 µL). The PCR product was extracted from a 2% agarose gel and puriﬁed using an AxyPrep DNA Gel Extraction Kit (Axygen Biosciences, Union City, CA, United States) according to the manufacturer’s instructions and quantiﬁed using a QuantiFluorTM ST ﬂuorometer (Promega, UnitedStates).

- 1. **Illumina MiSeq Sequencing**

Puriﬁed amplicons were pooled in equimolar amounts and paired-end sequenced (2 × 300) on an Illumina MiSeq platform (Illumina, San Diego, United States) according to standard protocols by Majorbio Bio-PharmTechnology Co., Ltd. (Shanghai,China). Processing of Sequencing Data The 16S rRNA sequencing data were processed using the Quantitative Insights Into Microbial Ecology (QIIME) platform (V.1.9.1)(7). The raw 16S rRNA reads were demultiplexed, quality ﬁltered by Trimmomatic and merged by FLASH with the following criteria: (i) The reads were truncated at any site that received an average quality score < 20 over a 50 bp sliding window. (ii) The primers were exactly attched, allowing a 2-nucleotide mismatch, and reads containing ambiguous bases were removed. (iii) Sequences with overlaps of longer than 10 bp were merged according to their overlap sequence. Operational taxonomic units (OTUs) were clustered with a 97% similarity cutoff using UPARSE (version7.1 http://drive5.com/uparse/), and chimeric sequences were identified and removed using UCHIME. The taxonomy of each 16S rRNA gene sequence was analyzed by the RDP Classifier algorithm (http://rdp.cme.msu.edu/) against the Silva 16S rRNA database (silva 132/16s bacteria) using a confidence threshold of 70%.(8, 9)

- 1. **Processing of sequencing data.**

The 16S rRNA sequencing data were processed using the Quantitative Insights Into Microbial Ecology (QIIME) platform (V.1.9.1)(10). The raw 16S rRNA reads were demultiplexed, quality filtered by Trimmomatic and merged by FLASH with the following criteria(8): (1) the 300 bp reads were truncated at any site receiving an average quality score <20 over a 50 bp sliding window, and the truncated reads shorter than 50 bp were discarded; (2) reads with exact barcode matching, 2 nucleotide mismatches in primers, or ambiguous characters were removed; and (3) only overlapping sequences longer than 10 bp were assembled according to their overlapping sequence. Reads that could not be assembled were discarded.

Operational taxonomic units (OTUs) were clustered with a 97% similarity cutoff using UPARSE (version 7.1, http://drive5.com/uparse/), and chimeric sequences were identified and removed using UCHIME(11, 12). The taxonomy of each representative OTU sequence was assigned by RDP Classifier (http://rdp.cme.msu.edu/) via comparison with the 16S rRNA database using a confidence threshold of 0.7.(13)

- 1. **Sequencing data analysis**

Operational taxonomic units (OTUs) were delineated at the cutoff of 97% using the USEARCH v.8.0(11). The protocol can be found on the website http:// drive5.com/usearch/manual/uparse_pipeline.html. The detailed procures were stated in our previous publication(14). Representative sequences for each OTU were built into a phylogenetic tree by FastTree and subjected to the RDP classifier (RDP database version 11.5, <http://rdp.cme.msu.edu/classifier/classifier.jsp>)(15) to determine the phylogeny with a bootstrap cut-off of 80%.The sequences of all the samples were downsized to 10,800 (1000 permutations) to match the difference in sequencing depth. α- andβ-diversity analyses were performed using Qiime v1.8.0(16). Shannon’s index, the observed OTUs, and Chao1 index were evaluated. A normalized OTU abundance table was used for the β-diversity analysis, including principal coordinate analysis (PCoA) based on Bray-Curtis, weighted UniFrac, and unweighted UniFrac distances. PERMANOVA was used to test for statistical significance between the groups using 9999 permutations. To calculate the variation explained by each of our collected host factors, we performed an Adonis test implemented in R. Each host factor was calculated according to its explanation rate, and P values were generated based on 9999 permutations.

- 1. **Microbial dysbiosis analysis**

To determine the degree of microbial dysbiosis, the microbial dysbiosis index was calculated as previously reported.(17, 18) Here, the microbial dysbiosis index was determined as the log of [total abundance of organisms increased in schizophrenia patients]/ [total abundance of organisms decreased in controls] for all samples.

- 1. **Microbial cluster generation using SparCC**

The OTUs shared by at least 20% among all the samples were considered key OTUs. The correlation among 274 key OTUs was calculated by the SparCC algorithm(19) with a bootstrap procedure repeated 100 times, and then correlation matrices were computed from the resampled data matrices. Once the bootstrapped correlation scores have been computed, only OTUs with correlation scores greater than 0.4 were classified into CAGs. Meanwhile, we threshold the P value at the desired cut-off <0.05. The correlation values were converted to a correlation distance (1-correlation value), and the OTUs were clustered using the Ward clustering algorithm via the R package WGCNA. Similar clusters were subsequently merged if the correlation between the CAG’s eigenvectors exceeded 0.8. The CAG network was visualized in Cytoscape (version 3.2.1). The similarities between any two network structures were measured by node closeness and shared correlations(20).

- 1. **Spearman multi-omic correlation analysis**

Spearman correlations between CAGs, serum metabolite modules and clinical parameters were calculated using R, and both differential abundances of CAGs and CAD-associated metabotypes were tested by the Wilcoxon rank sum test. Wherever mentioned, the Benjamini-Hochberg method was used to control the FDR. The visual presentation of multiple omics correlations was performed using the R. ggplot2 package.

1. **Untargeted metabolomics study**
   1. **Metabolite Extraction from fecal and serum**

50mg fecal sample or 100ul serum sample were accurately weighed, and the metabolites extracted using a 400 µL methanol: water (4:1, v/v) solution. The mixture was allowed to settle at -20^o^C and treated by high throughput tissue crusher Wonbio-96c (Shanghai wanbo biotechnology co., LTD) at 50 Hz for 6 min, then followed by vortex for 30s and ultrasound at 40 kHz for 30 min at 5^o^C. The samples were placed at -20^o^C for 30min to precipitate proteins. After centrifugation at 13000g at 4^o^C for 15min, the supernatant was carefully transferred to sample vials for LC-MS/MS analysis.

- 1. **Quality control sample**

As a part of the system conditioning and quality control process, a pooled quality control sample (QC) was prepared by mixing equal volumes of all samples. The QC samples were disposed and tested in the same manner as the analytic samples. It helped to represent the whole sample set, which would be injected at regular intervals (every 8 samples of fecal sample and 10 samples of serum sample) in order to monitor the stability of the analysis.

- 1. **UPLC-MS/MS analysis.**

Chromatographic separation of the metabolites was performed on a ExionLC^TM^AD system (AB Sciex, USA) equipped with an ACQUITY UPLC BEH C18 column (100 mm × 2.1 mm i.d., 1.7 µm; Waters, Milford,USA). The mobile phases consisted of 0.1% formic acid in water with formic acid (0.1%) (solvent A) and 0.1% formic acid in acetonitrile: isopropanol (1:1, v/v) (solvent B). The solvent gradient changed according to the following conditions: from 0 to 3 min, 95% (A): 5% (B) to 80% (A): 20% (B); from 3 to 9 min, 80% (A): 20% (B) to 5% (A): 95% (B); from 9 to 13 min, 5% (A): 95% (B) to 5% (A): 95% (B): from 13 to 13.1 min, 5% (A): 95% (B) to 95% (A): 5% (B), from 13.1 to 16 min, 95% (A): 5% (B) to 95% (A): 5% (B) for equilibrating the systems. The sample injection volume was 20 uL and the flow rate was set to 0.4 mL/min. The column temperature was maintained at 40^o^C. During the period of analysis, all these samples were stored at 4^o^C.

The UPLC system was coupled to a quadrupole-time-of-flight mass spectrometer (Triple TOF^TM^5600+, AB Sciex, USA) equipped with an electrospray ionization (ESI) source operating in positive mode and negative mode. The optimal conditions were set as followed: source temperature, 500^o^C; curtain gas (CUR), 30 psi; both Ion Source GS1 and GS2, 50 psi; ion-spray voltage floating (ISVF), -4000V in negative mode and 5000V in positive mode, respectively; declustering potential, 80V; a collision energy (CE), 20-60V rolling for MS/MS. Data acquisition was performed with the Data Dependent Acquisition (DDA) mode. The detection was carried out over a mass range of 50-1000 m/z.

- 1. **Data preprocessing and annotation**

After UPLC-TOF/MS analyses, the raw data were imported into the Progenesis QI 2.3 (Nonlinear Dynamics, Waters, USA) for peak detection and alignment. The preprocessing results generated a data matrix that consisted of the retention time (RT), mass-to-charge ratio (m/z) values, and peak intensity. Metabolic features detected at least 80 % in any set of samples were retained. After filtering, minimum metabolite values were imputed for specific samples in which the metabolite levels fell below the lower limit of quantitation and each Metabolic feature were normalized by sum. The internal standard was used for data QC (reproducibility), Metabolic features which the relative standard deviation (RSD) of QC>30% were discarded. Following normalization procedures and imputation, statistical analysis was performed on log transformed data to identify significant differences in metabolite levels between comparable groups. Mass spectra of these metabolic features were identified by using the accurate mass, MS/MS fragments spectra and isotope ratio difference with searching in reliable biochemical databases as Human metabolome database (HMDB) (http://www.hmdb.ca/) and Metlin database (https://metlin.scripps.edu/). Concretely, the mass tolerance between the measured m/z values and the exact mass of the components of interest was ±10ppm. For metabolites having MS/MS confirmation, only the ones with MS/MS fragments score above 30 were considered as confidently identified. Otherwise, metabolites had only tentative assignments.

- 1. **Multivariate statistical analysis**

A multivariate statistical analysis was performed using ropls (Version1.6.2, http://bioconductor.org/packages/release/bioc/html/ropls.html) R package from Bioconductor on Majorbio Cloud Platform (https://cloud.majorbio.com). Principle component analysis (PCA) using an unsupervised method was applied to obtain an overview of the metabolic data, general clustering, trends, or outliers were visualized. All of the metabolite variables were scaled to unit-variances prior to conducting the PCA. Orthogonal partial least squares discriminate analysis (OPLS-DA) was used for statistical analysis to determine global metabolic changes between comparable groups. All of the metabolite variables were scaled to pareto Scaling prior to conducting the OPLS-DA. The model validity was evaluated from model parameters R2 and Q2, which provide information for the interpretability and predictability, respectively, of the model and aviod the risk of over-fitting. Variable importance in the projection (VIP) were calculated in OPLS-DA model. p values were estimated with paired Student’s t-test on Single dimensional statistical analysis.

- 1. **Differential metabolites analysis**

Statistically significant among groups were selected with VIP value more than 1 and p value less than 0.05. For feces, 1,378 features at ESI+ ion mode and 1,246 features at ESI− ion mode were tested in this experiment. And for serum, 531 features at ESI+ ion mode and 336 features at ESI− ion mode were detected. Differential metabolites among two groups were summarized, and mapped into their biochemical pathways through metabolic enrichment and pathway analysis based on database search (KEGG, http://www. genome.jp/kegg/). These metabolites can be classified according to the pathways they involved or the functions they performed. Enrichment analysis was usually to analyze a group of metabolites in a function node whether appears or not. The principle was that the annotation analysis of a single metabolite develops into an annotation analysis of a group of metabolites. scipy.stats (Python packages) ( https://docs.scipy.org/doc/scipy/ ) was exploited to identify statistically significantly enriched pathway using Fisher’s exact test.

- 1. **Clustering of co-abundant fecal and serum metabolites.**

Clusters of co-abundant serum metabolites were identified using the R package WGCNA(21), along with official tutorials (<https://horvath.genetics.ucla.edu>). Signed, weighted metabolite co-abundance correlation networks were calculated for all examined individuals. A scale-free topology criterion was used to choose the soft threshold β = 14 for fecal and serum metabolites correlations. Clusters were identified with the dynamic hybrid tree-cutting algorithm using a deepSplit of 4.(22) The metabolites were collectively termed metabolite clusters.

1. **Inflammatory biomarker assays**

The concentration of serum cytokines (CCL4, Granzyme B, HGF, IFN-alpha, IFN-beta, IFN-gamma, IL-1 beta, IL-1ra, IL-2, IL-4, IL-5, IL-6, IL-7, IL-8, IL-10, IL-12 p40, IL-17, IL-18, SCGF, TNF-alpha) were analyzed employing xMAPtechnology, following the manufacturer’s instructions (LXSAHM-20, R&D Systems Inc.; HGAMMAG-301K-03, Merck Millipore). Briefly, serum samples were incubated with antibodies conjugated to microspheres for 2 h at room temperature, then, the samples were incubated with biotinylated antibodies for 1 h, followed by incubation with streptavidin-phycoerythrin fluorescent conjugate (SA-PE) for 30 min. The Luminex® 200TM instrument (Luminex Corp.) detected the intensity of the signal for each microsphere added to the protein samples. The concentration of each analyte was calculated against standard curve regression, using Luminex® xPONENT software.

**Reference**

1. Wang R, Lai XH, Liu X, Li YJ, Chen C, Li C, et al. Brain magnetic resonance-imaging findings of anti-N-methyl-D-aspartate receptor encephalitis: a cohort follow-up study in Chinese patients. J Neurol. 2018;265(2):362-9. doi: 10.1007/s00415-017-8707-5. Epub 2017 Dec 16.

2. Liu X, Yan B, Wang R, Li C, Chen C, Zhou D, et al. Seizure outcomes in patients with anti-NMDAR encephalitis: A follow-up study. Epilepsia. 2017;58(12):2104-11. doi: 10.1111/epi.13929. Epub 2017 Nov 3.

3. Liu X, Zhang L, Chen C, Gong X, Lin J, An D, et al. Long-term cognitive and neuropsychiatric outcomes in patients with anti-NMDAR encephalitis. Acta Neurol Scand. 2019;140(6):414-21. doi: 10.1111/ane.13160. Epub 2019 Oct 10.

4. Wang R, Guan HZ, Ren HT, Wang W, Hong Z, Zhou D. CSF findings in patients with anti-N-methyl-D-aspartate receptor-encephalitis. Seizure. 2015;29:137-42.(doi):10.1016/j.seizure.2015.04.005. Epub Apr 22.

5. Wang W, Li JM, Hu FY, Wang R, Hong Z, He L, et al. Anti-NMDA receptor encephalitis: clinical characteristics, predictors of outcome and the knowledge gap in southwest China. Eur J Neurol. 2016;23(3):621-9. doi: 10.1111/ene.12911. Epub 2015 Nov 12.

6. Tang S, Zheng C, Chen M, Du W, Xu X. Geobiochemistry characteristics of rare earth elements in soil and ground water: a case study in Baotou, China. Sci Rep. 2020;10(1):11740. doi: 10.1038/s41598-020-68661-4.

7. Kuczynski J, Stombaugh J, Walters WA, González A, Caporaso JG, Knight R. Using QIIME to analyze 16S rRNA gene sequences from microbial communities. Curr Protoc Bioinformatics. 2011;Chapter(10):Unit 10.7.. doi: .1002/0471250953.bi1007s36.

8. Magoč T, Salzberg SL. FLASH: fast length adjustment of short reads to improve genome assemblies. Bioinformatics. 2011;27(21):2957-63. doi: 10.1093/bioinformatics/btr507. Epub 2011 Sep 7.

9. Segata N, Izard J, Waldron L, Gevers D, Miropolsky L, Garrett WS, et al. Metagenomic biomarker discovery and explanation. Genome Biol. 2011;12(6):R60. doi: 10.1186/gb-2011-12-6-r60.

10. Kuczynski J, Stombaugh J, Walters WA, Gonzalez A, Caporaso JG, Knight R. Using QIIME to analyze 16S rRNA gene sequences from microbial communities. Curr Protoc Bioinformatics. 2011;Chapter 10:Unit 10 7.

11. Edgar RC. UPARSE: highly accurate OTU sequences from microbial amplicon reads. Nat Methods. 2013;10(10):996-8. doi: 10.1038/nmeth.2604. Epub 013 Aug 18.

12. Edgar RC, Haas BJ, Clemente JC, Quince C, Knight R. UCHIME improves sensitivity and speed of chimera detection. Bioinformatics. 2011;27(16):2194-200. doi: 10.1093/bioinformatics/btr381. Epub 2011 Jun 23.

13. Navas-Molina JA, Peralta-Sánchez JM, González A, McMurdie PJ, Vázquez-Baeza Y, Xu Z, et al. Advancing our understanding of the human microbiome using QIIME. Methods Enzymol. 2013;531:371-444.(doi):10.1016/B978-0-12-407863-5.00019-8.

14. Zhang Q, Wu Y, Wang J, Wu G, Long W, Xue Z, et al. Accelerated dysbiosis of gut microbiota during aggravation of DSS-induced colitis by a butyrate-producing bacterium. Sci Rep. 2016;6:27572.(doi):10.1038/srep27572.

15. Wang Q, Garrity GM, Tiedje JM, Cole JR. Naive Bayesian classifier for rapid assignment of rRNA sequences into the new bacterial taxonomy. Appl Environ Microbiol. 2007;73(16):5261-7. doi: 10.1128/AEM.00062-07. Epub 2007 Jun 22.

16. Caporaso JG, Kuczynski J, Stombaugh J, Bittinger K, Bushman FD, Costello EK, et al. QIIME allows analysis of high-throughput community sequencing data. Nat Methods. 2010;7(5):335-6. doi: 10.1038/nmeth.f.303. Epub 2010 Apr 11.

17. Gevers D, Kugathasan S, Denson LA, Vázquez-Baeza Y, Van Treuren W, Ren B, et al. The treatment-naive microbiome in new-onset Crohn's disease. Cell Host Microbe. 2014;15(3):382-92. doi: 10.1016/j.chom.2014.02.005.

18. Xu R, Wu B, Liang J, He F, Gu W, Li K, et al. Altered gut microbiota and mucosal immunity in patients with schizophrenia. Brain Behav Immun. 2020;85:120-127.(doi):10.1016/j.bbi.2019.06.039. Epub Jun 27.

19. Wishart DS, Feunang YD, Marcu A, Guo AC, Liang K, Vázquez-Fresno R, et al. HMDB 4.0: the human metabolome database for 2018. Nucleic Acids Res. 2018;46(D1):D608-D17. doi: 10.1093/nar/gkx89.

20. Wang J, Zheng J, Shi W, Du N, Xu X, Zhang Y, et al. Dysbiosis of maternal and neonatal microbiota associated with gestational diabetes mellitus. Gut. 2018;67(9):1614-25. doi: 10.136/gutjnl-2018-315988. Epub 2018 May 14.

21. Langfelder P, Horvath S. WGCNA: an R package for weighted correlation network analysis. BMC Bioinformatics. 2008;9:559.(doi):10.1186/471-2105-9-559.

22. Langfelder P, Zhang B, Horvath S. Defining clusters from a hierarchical cluster tree: the Dynamic Tree Cut package for R. Bioinformatics. 2008;24(5):719-20. doi: 10.1093/bioinformatics/btm563. Epub 2007 Nov 16.

**II. eFigures**


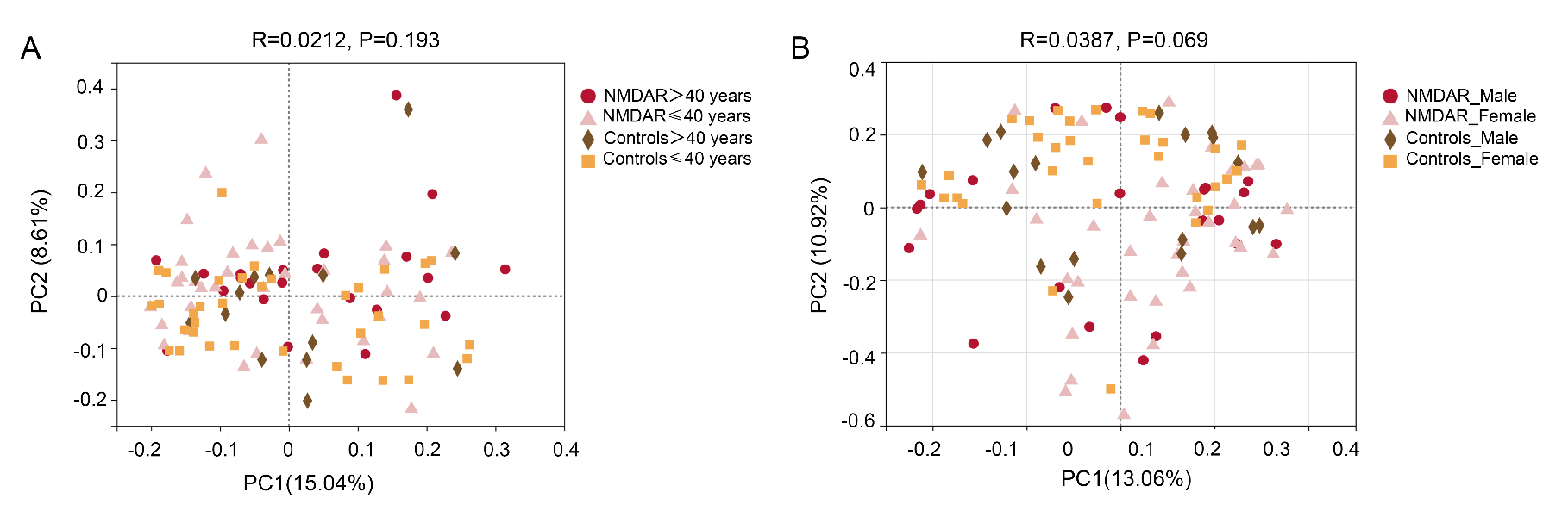


**eFigure 1: Validation of the differences in gender and age based on gut microbiome beta diversity.** Beta diversity indexes based on UniFrac analysis. (A) R2 and statistical test in permutational multivariate analysis of variance between younger healthy controls (age more than 40 years) and older HCs (age more than 40 years) and between younger NMDAR encephalitis patients and older patients. (B) R2 and statistical test in permutational multivariate analysis of variance between female healthy controls and male healthy controls and between female NMDAR encephalitis patients and male patients. UniFrac distance was based on the 16S data.


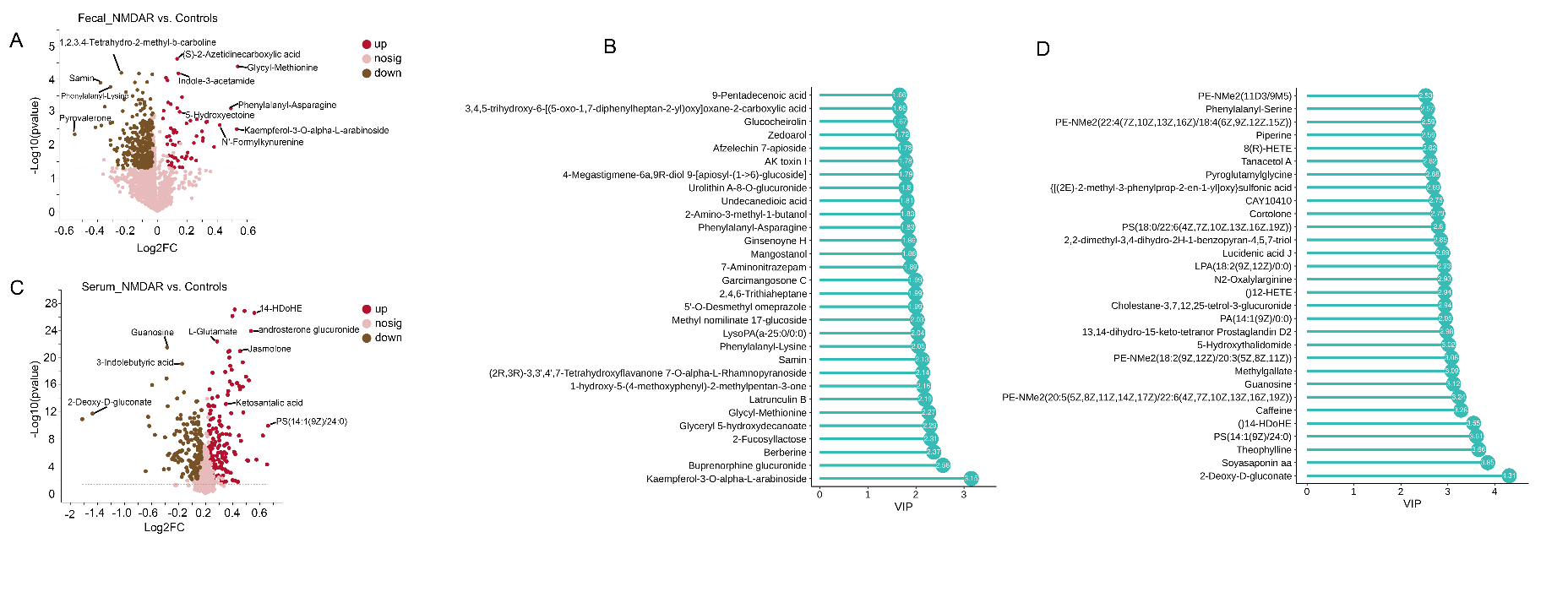


**eFigure 2: Fecal and serum metabolites differential NMDAR encephalitis case and healthy controls.** (A) Volcano plot of 298 adjusted PLS-DA -selected significant fecal metabolites. X-axis represents the log (fold-change), fold-change is the relative abundance of each metabolite between NMDAR encephalitis case versus healthy control groups. Y-axis represents the statistical significance adjusted p-value (Padj) for multiple testing with Benjamini-hochberg correction. Node color indicates the significant difference in case versus control groups: red, upregulated in NMDAR encephalitis cases, brown, downregulated in cases; gray, no significant difference. The horizontal red line corresponds to an adjused p-value cutoff of 0.05 with Benjamini-hochberg correction. (B) The variable importance in projection (VIP) score plot of the top 30 PLS-DA fecal metabolites, which most significantly separate disease cases from controls. A total of 177 metabolites had VIP >1. (C) Volcano plot of 278 unadjusted Wilcoxon-selected significant serum metabolites. (D) (VIP) score plot of the top 30 PLS-DA fecal metabolites. A total of 267 metabolites had VIP >1.
